# Experimental data for the synthesis of a new dimeric prodelphinidin gallate

**DOI:** 10.1016/j.dib.2016.06.023

**Published:** 2016-06-22

**Authors:** Natércia Teixeira, Nuno Mateus, Victor de Freitas

**Affiliations:** QUINOA-LAQV-REQUIMTE, Departamento de Química e Bioquímica, Faculdade de Ciências, Universidade do Porto, Rua do Campo Alegre, 687, 4169-007 Porto, Portugal

**Keywords:** Prodelphinidins, Synthesis, Hydrogenation

## Abstract

This data article contains raw and processed data related to research published in Teixeira et al. (2016) [1]. Here we introduce data acquired from the synthesis of a prodelphinidin dimer gallate. All synthesis steps are described and a dataset for the removal of the protecting on prodelphinidin synthesis is presented. With hydrogenolysis *in situ* with triethylsilane the hydrogen required is produced and used at the same time, making the reaction possible without resorting to bottled hydrogen. Full NMR and HPLC-ESI-MS analysis data is also provided.

**Specifications Table**

**Table:** 

Subject area	Chemistry
More specific subject area	Food Chemistry
Type of data	Table, figures
How data was acquired	NMR (Bruker Avance 400 spectrometer), mass spectroscopy (Finnigan Surveyor Plus HPLC system fitted with a PDA Plus detector, an autosampler Plus and a LC quaternary pump plus coupled to a Finnigan LCQ Deca XP Plus mass detector equipped with a ESI source and an ion trap quadrupole equipped with an atmospheric pressure ionization (API) source).
Data format	Analyzed
Experimental factors	Synthesis procedure of a prodelphinidin dimer gallate starting from the two constitutive monomers.
Experimental features	Starting constitutive monomer protection with benzyl groupsDDQ oxidation at C4 of the upper monomer with benzyl alcohol as the nucleophile and DMAP as nucleophilic catalyst.Condensation of the two constitutive monomersHydrogenolysis *in situ* with triethylsilaneNMR and HPLC-ESI-MS data analysis
Data source location	Oporto, Portugal
Data accessibility	Data are available with the article

## **Value of the data**

• The data for a simple method for prodelphinidin gallate synthesis with hydrogenolysis *in situ* is presented;
• Full NMR analysis data is included for easy identification of the intermediate and final compounds;
• This synthesis strategy may be applied to other proanthocyanidins.

## 1 Data

**Fig. 1:**
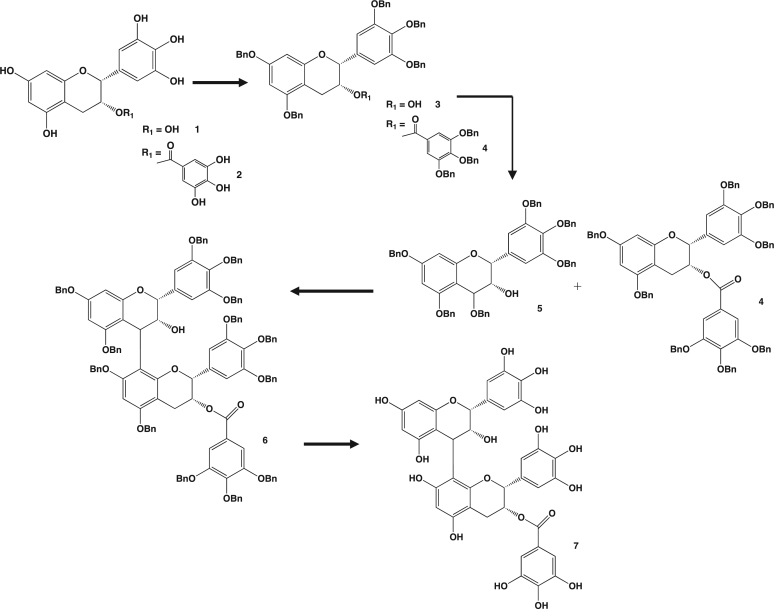
Synthesis of prodelphinidin epigallocatechin-(4β→8)-epigallocatechin gallate (EGC-EGCG).

The data here presented describes the synthesis procedure and analysis of epigallocatechin-(4β→8)-epigallocatechin gallate (EGC-EGCG), a prodelphinidin dimer (Fig. 1) [1]. The synthesis procedure starts from the two constitutive monomers: (-)-Epigallocatechin (EGC) and (-)-epigallocatechin gallate (EGCG), and includes a new approach to the removal of the protecting groups.

## 2 Experimental design, materials and methods

### 2.1 Materials

(-)-Epigallocatechin (EGC) and (-)-epigallocatechin gallate (EGCG) were purchased from Biopurify Phytochemicals Ltd. (Sichuan, China).

### 2.2 Methods

#### 2.2.1 Benzylation of monomeric flavan-3-ols

To a stirred solution of (-)-EGC **1** and (-)-EGCG **2** in dry DMF, under argon, was added potassium carbonate (K_2_CO_3_) (10 eq for **1**; 17.6 eq for **2**) and BnBr (7.7 eq for **1**; 13.6 eq for **2**). The solution was stirred at 0 °C for 2 h and left at room temperature for 72 h for **1** and 24 h for **2**. The mixture was extracted with ethyl acetate and water, dried over Na_2_SO_4_, filtered and concentrated. The crude product was purified with silica gel column chromatography (dichloromethane (CH_2_Cl_2_) for **3** and hexane/ EtOAc 2:1 for **4**).

#### 2.2.2 Benzylation at C4

To a solution of EGC5Bn 3 and benzyl alcohol (BnOH) (10.3 eq) in CH_2_Cl_2_ was slowly added, at 0 °C, 2,3-dichloro-5,6-dicyano-1,4-benzoquinone (DDQ) (2.3 eq). After reacting overnight at room temperature, it was added 4-dimethylaminopyridine (DMAP) (2,4 eq) at 0 °C and left to react for 30 min. Then the mixture was filtered and washed with water and brine, dried over Na_2_SO_4_, filtered and concentrated. The crude product was purified with silica gel column chromatography with EtOAc 2:1 as eluent to afford EGC5Bn(Bn) 5.

#### 2.2.3 Condensation

EGC5Bn(Bn) **5** and EGCG8Bn **4** (4 eq) were dissolved in CH_2_Cl_2_ and trimethylsilyl trifluoromethanesulfonate (TMSOT_f_) (0.5 M solution in CH_2_Cl_2_, 1.5 eq) was added dropwise at −78°C. Therefore the proper time of reaction was tested by following each reaction by TLC. At first the solutions were left to react for 5 min, following the method described by Krohn et al. [2]. However, after that period of time and after checking the reaction products by TLC, it was observed that the upper unit (and limiting reagent) EGC5Bn(Bn) was still present in good quantity. Thereby, the solution was left stirring for 90 min (reaching −22°C), and left to reach 0 °C for 3 h 30 min. The reactions were then quenched by addition of saturated aqueous sodium bicarbonate (Na_2_HCO_3_) (1 mL). The mixture was extracted with chloroform and the organic phase was washed with water and brine, dried over Na_2_SO_4_, filtered and concentrated. The crude product was purified with silica gel column chromatography with CH_2_Cl_2_ as eluent to afford EGC-EGCG13Bn **6**.

#### 2.2.4 Hydrogenolysis *in situ*

To a stirred solution of EGC-EGCG13Bn 25 under argon and Pd/C 10% in MeOH (2–3 mL) was added neat triethylsilane (TES) (10 mmol for each removing group) [3]. A few drops of THF were added to dissolve the reagents. When the reaction was complete (TLC), each mixture was filtered through a 0.20 μm PET Chromafil® syringe filter and the solvent was evaporated under vacuum.

### 2.3 Experimental design

In order to confirm the presence of the desired products on each synthesis step, NMR and HPLC-ESI-MS analysis were performed.

#### 2.3.1 5,7,3′,4′,5′-Penta-O-benzylepigallocatechin (EGC5Bn) (3)

Amorphous buff solid, yield 83.9%. ESI-MS found [M+H]^+^: 757. ^1^H NMR (CDCl_3_) δ/ppm: 2.98 (H4, ABX), 4.20 (H3, br), 4.88 (H2, s), 5.01, 5.06, 5.13 (OCH_2_Bn, s), 6.28 (H6 and H8, s), 6.81 (H2′ and H4′, s), 7.23–7.43 (H-Ar, m); ^13^C NMR (CDCl_3_) δ/ppm: 28.12 (C4), 66.30 (C3), 69.88, 70.08, 71.23 (OCH_2_Bn), 78.63 (C2), 94.17 (C8), 94.75 (C6), 101.14 (C4a), 106.16 (C2′ and C6′), 126.90–138.20 (C-Ar), 140.99 (C4′), 152.96 (C3′ and C5′), 155.21 (C5), 158.27 (C8a), 158.78 (C7).

#### 2.3.2 4,5,7,3′,4′,5′-Hexa-O-benzylepigallocatechin ((EGC5Bn)Bn) (5)

Amorphous buff solid, yield 98.3%. ESI-MS found [M+H]^+^: 863. ^1^H NMR (CDCl_3_) δ/ppm: 3.93 (H3, m), 4.22 (H4, m), 4.73 (OCH_2_Bn, d, *J*=1.76 Hz), 5.04 (OCH_2_Bn, d, *J*=4.53 Hz), 5.13 (OCH_2_Bn, s), 5.22 (H2, s), 6.28 (H8, d, *J*=2.26 Hz), 6.31 (H6, d, *J*=2.21 Hz), 6.77 (H2′ and H6′, s), 7.25-7.44 (H-Ar); ^13^C NMR (CDCl_3_) δ/ppm: 68.91 (C4), 70.18 (C3), 70.46, 70.57, 71.44, 72.17, 75.11, 75.38 (OCH_2_Bn), 94.21 (C6), 94.52 (C8), 106.32 (C2′ and C6′), 127.66-129.09 (C-Ar), 133.44 (C1′), 134.53, 136.46, 136.77, 137.11, 137.92, 138.34 (C-Ar), 139.04 (C4′), 153.13 (C5′), 156.04 (C3′), 159.90 (C5), 160.77 (C7).

#### 2.3.3 5,7,3′,4′,5′-Penta-O-benzyl-3-O-(3,4,5-tri-O-benzylgalloyl)epigallocatechin (EGCG8Bn) (4)

Amorphous yellow solid, yield 97.2%. ESI-MS found [M+H]^+^: 1179. ^1^H NMR (CDCl_3_) δ/ppm: 3.22 (H4, ABX), 4.25 (H3, br), 5.05 (H2, s) 5.10-5.16 (OCH_2_Bn), 6.87 (H2′′ and H6′′, br s), 6.54 (H2′ and H6′, s), 7.24-7.51 (H-Ar, m); ^13^C NMR (CDCl_3_) δ/ppm: 26.32 (C4), 68.55 (C3), 70.16, 70.30, 71.20, 71.33, 75.16, 75.25 (OCH_2_Bn), 78.03 (C2), 94.23 (C6), 94.92 (C8), 101.25 (C4a), 106.94 (C2′ and C6′), 109.29 (C2′′ and C6′′), 127.35-128.69 (C-Ar), 130.01 (C1′), 136.57 (C1′′), 136.93-138.61 (C-Ar), 142.90 (C4′′), 152.58 (C3′ and C5′), 153.07 (C3′′ and C5′′), 133.40 (C4′), 155.84 (C8a), 158.19 (C5), 159.04 (C7), 164.94 (CO).

#### 2.3.4 5,7,3′,4′,5′-Penta-O-benzylepigallocatechin(4→8)5,7,3′,4′-Tetra-O-benzylcatechin (EGC-EGCG13Bn) (6)

Yellow oil, yield 92.1%. ESI-MS found [M+H]^+^: 1935. ^1^H NMR (CDCl_3_) δ/ppm: 4.12 (H-4C, br), 4.39 (H-3C, br), 4.60 (H-2C, d, *J*=1.88 Hz), 4.73–5.00 (OCH_2_Bn, m), 5.44 (H-3F, d, *J*=5.14 Hz), 5.81 (H-2F, d, *J*=2.22 Hz), 6.15 (H-6F, s), 6.23 (H-8F, s), 6.42 (H-2′C, H-6′C, H-2′F and H-6′F, s), 6.97 (H-2G and H-6G, s), 7.04–7.24 (H-Ar, m); ^13^C NMR (CDCl_3_) δ/ppm: 15.51 (C-4F and C-4C), 19.72 (C-3F and C3), 20.81, 21.41 (OCH_2_Bn), 28.87 (C-2C and C-2F), 71.41 (C-2′F and C-2′C), 75.00 (C-8F, C-2G and C-6G), 125.73, 126.00, 127.76, 128.06, 128.82 (C-Ar), 127.05 (C-1G), 128.21 (C-1′F, C-4′F, C-1′C and C-4′C), 129.50 (C-4G), 129.74 (C-3′C, C-5′C, C-3′F, C-5′F, C-3G and C-5G), 134.50 (C-8aF), 136.34 (C-8aC), 137.53 (C-5F, C-7F and C-7C), 144.07 (C-5C), 153.06 (CO).

#### 2.3.5 Epigallocatechin(4→8)epigallocatechin gallate (EGC-EGCG) (7)

**Fig. 2:**
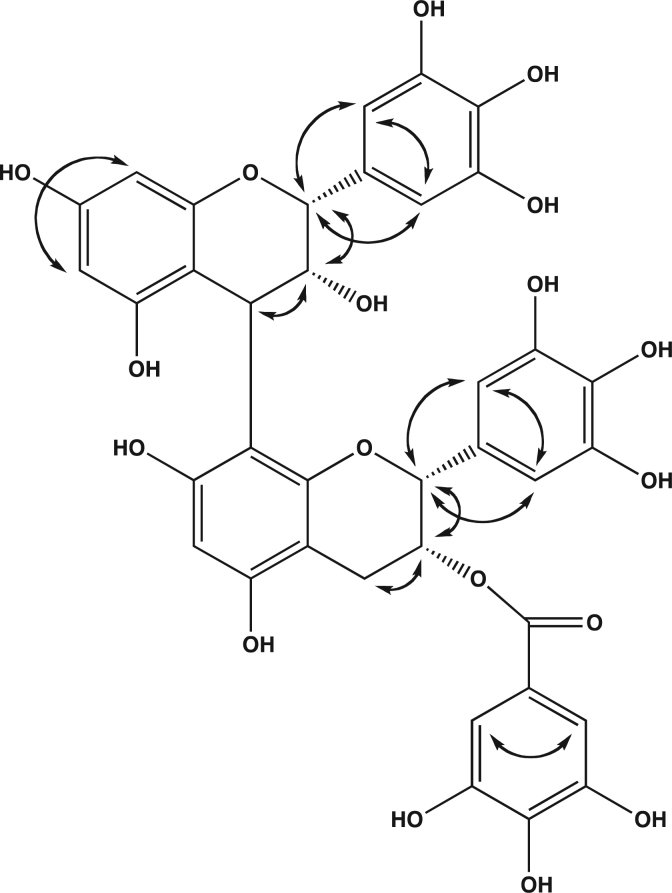
COSY correlations of EGC-EGCG **7**.

White powder, yield 6%. ESI-MS found [M+H]^-^: 761. The ^1^H and ^13^C-NMR chemical shifts are reported in Table 1 and COSY correlations in Fig. 2.

## Figures and Tables

**Fig. Transparency document1:**
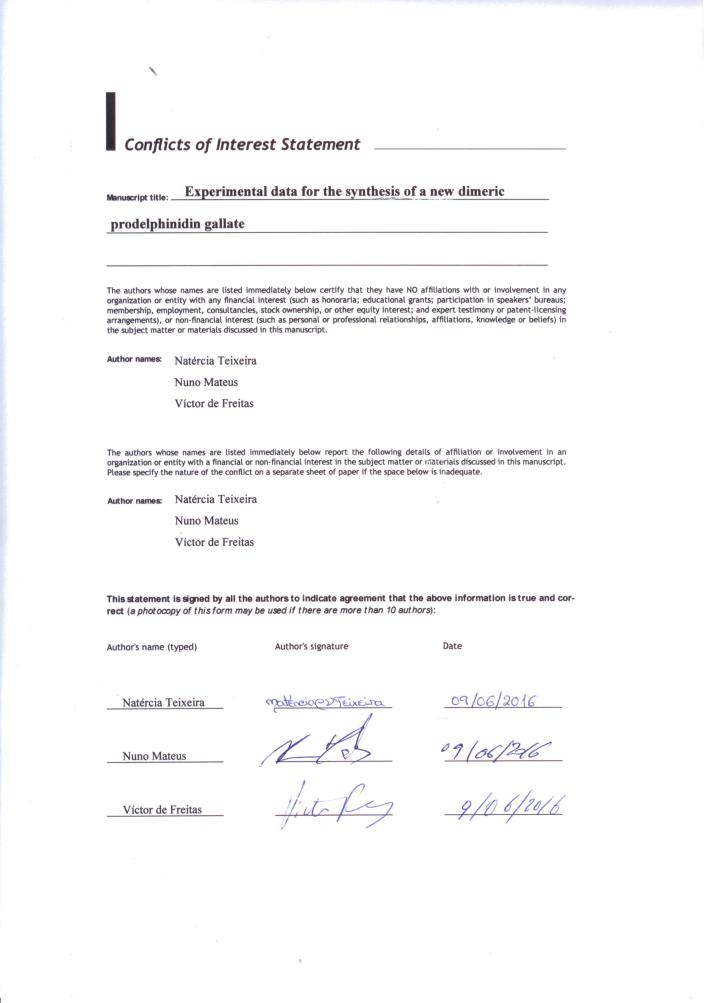


**Table 1:** ^1^H and ^13^C-NMR data and HMBC and HSQC correlations of EGC-EGCG **7**, determined in MeOD.

**Position**	**δ**^**1**^**H (ppm) J (Hz)**	**δ**^13^**C (ppm)**	**HMBC**	**HSQC**	**NOESY**
2C	5.20; br	79.1	H-2′C; H-6′C	H-2C	–
3C	4.58; tr, 3.31	69.2	H-2C	–	H-4C; H-2C; H-6F
4C	4.19; d, 3.14	29.4	H-2C	–	–
4aC	–	102.5	–	–	–
5C	–	154.9	–	–	–
6C	6.08; br d	98.8	–	H-6C	–
7C	–	150.1-152.3	–	–	–
8C	6.10; br d	98.8	–	H-8C	–
8aC	–	150.1-152.3	–	–	–
1′C	–	130.5	H-2′C	–	–
2′C	6.77; s	105.9	H-2C; H-4C; H-6′C	H-2′C	–
3′C	–	145.2	H-2′C	–	–
4′C	–	133.2	H-2′C	–	–
5′C	–	145.2	H-6′C	–	–
6′C	6.77; s	105.9	H-2C; H-4C; H-2′C	H-6′C	–
2 F	5.71; br d	79.1	H-4F; H-2′F; H-6′F	–	–
3 F	5.68; br	66.7	H-4F	H-3F	–
4 F	2.99; dd, 17.8 3.09; dd, 17.5	22.8	–	H-4F	H-2C; H-6C
4 aF	–	102.5	H-4F	–	–
5 F	–	154.9	–	–	–
6 F	6.21; s	98.8	–	H-6F	–
7 F	–	150.1-152.3	–	–	–
8 F	–	128.0	–	–	–
8 aF	–	150.1-152.3	–	–	–
1′F	–	130.5	H-2′F	–	–
2′F	6.77; s	100.7	H-6′F	–	–
3′F	–	145.2	H-2′F	–	–
4′F	–	133.2	H-2′F	–	–
5′F	–	145.2	H-6′F	–	–
6′F	6.77; s	100.7	H-2′F	–	–
1G	–	119.7	–	–	–
2G	6.85; s	108.9	–	H-2G	–
3G	–	139.6	H-2G	–	–
4G	–	141.8	–	–	–
5G	–	139.6	H-6G	–	–
6G	6.85; s	108.9	–	H-6G	–
C=O	–	166.5	H-2G; H-6G	–	–
OH	4.99; br	–	–	–	–
3C–OH	4.04; br	–	–	–	–
